# The value of Life Sciences in an Integrated Curriculum: A Reflective Perspective of studying two Life Sciences Degrees a decade apart and the challenges faced in professional life

**DOI:** 10.15694/mep.2017.000117

**Published:** 2017-07-03

**Authors:** Noamaan Wilson-Baig

**Affiliations:** 1 Tameside General Hospital

**Keywords:** Life Sciences, Basic Science, Integrated Curriculum, Research, Medical Education

## Abstract

This article was migrated. The article was marked as recommended.

In a recent MedEdPublish article by Keenan and Jennings (2017), I was interested in why some Life Sciences are under-represented in the wider medical literature. The article states anatomy to be the dominant discipline within medical schools and describes a close link between anatomy and educational research with the presence of an “established medical education research community and social media community linked to anatomy”. Unfortunately, this does not appear to be the case with other scientific disciplines. In my career, I have been fortunate to have studied both Pharmacy and Medicine as an undergraduate. I also possess an MSc in Clinical Pharmacy and am undertaking an MSc in Clinical Research. I have seen at first-hand how integral the Life Sciences are in the practice of Pharmacy and Medicine. It is therefore necessary that, like anatomy, other Life Sciences should enjoy a similar commitment to “maintaining a scholarly approach to teaching and learning ( Keenan and Jennings, 2017). Currently, I am an Academic Clinical Registrar in Anaesthesia working in the North-West. I am part funded by the National Institute for Health Research (NIHR). Considering my background, I would like to share a personal reflection of my experience of undergraduate training and professional life. I will compare the undergraduate teaching styles I experienced whilst studying for my Pharmacy and Medicine degrees. I will also describe the challenges I faced and the moments of enlightenment I felt when I chose to embark on a career in academia with clinical practice.

## Personal Reflection

As a Pharmacy undergraduate, a large proportion of my curriculum consisted of basic science. We had lectures and practical sessions in organic chemistry, inorganic chemistry, pharmacology, pharmacognosy and pharmaceutics. I remember conducting basic scientific investigations that involved organ bath experiments. The aim was to learn the pharmacological effects of drugs on alpha and beta receptors in muscle. I also remember making creams and suppositories from basic ingredients. This is something that a large proportion of qualified pharmacists will not get involved in unless they are working in industry or an aseptics department within a hospital. It was not until my final year that I eventually sampled a flavour of what working in a pharmacy was like, and even then this experience formed a minor part of the course. Once I had successfully obtained my degree in 1994, I took up a pre-registration post at Luton and Dunstable Hospital. I quickly realised that the job I was doing required me to attain new “task orientated” skills that I had not been taught at university. Additionally, I was learning to apply the knowledge gained at university to real life. I felt overwhelmed and unprepared for working life as a pharmacist. At the time I pondered the value of studying basic science on my pharmacy course, especially areas such as inorganic chemistry. Research was a distant entity as I was coming to grips with the realities of the “task driven environment” I was working in.

I first experienced meaningful research in 1998 when I undertook an MSc in Clinical Pharmacy. For my research project, I conducted an open-labelled retrospective study of topiramate as add-on therapy in paediatric patients suffering from epilepsy. Its efficacy and tolerability were examined as well as the effective dose ranges for a particular seizure type. Any correlation between age and drug dose was investigated. For the first time, I appreciated the value of basic science with regards to physiology, pathology, pharmacology and paediatrics. I remember enjoying the experience and wondered whether pharmacy undergraduates could get the same buzz I did from performing research projects as opposed to learning via lectures of subjects in isolation. At least this method allowed me to learn elements of all the sciences pertaining to the project but also how the subjects related to each other. I felt I had gained a global understanding of a subject that I knew very little about prior to starting the research project. I also appreciated how patients suffering from epilepsy coped and managed their day-day living. Admittedly, I had to be self-driven and revisit elements of basic science learnt at undergraduate level and learn other sciences I had not, e.g. sociology and psychology.

As a result of this experience, I wanted to delve further into research. As I had already experienced research in the clinical setting, I wanted to go explore research within the laboratory setting. At this point, I was a newly appointed Haematology / Oncology Pharmacist with little experience or knowledge in the area. I thought it pertinent to follow a similar strategy to before and undertook a research project within the field of haematology. In this project, I was involved in investigating the cellular and molecular mechanisms of stress-induced premature senescence in chronic lymphocytic leukaemia cells. It was thought exploiting this mechanism could be used to halt cell proliferation in myeloma cell lines. I took unpaid leave, funded myself and undertook a certificate in Immunology with Manchester University to consolidate my learning “on the job”. Once again, I saw first-hand how basic science was involved in the identification of novel biochemical pathways. It also provided me with first-hand experience in learning laboratory techniques whilst acquiring skills in molecular biology and biochemistry applied to muscle cells and tissues. Working as a hospital pharmacist in this area allowed me to see how all the pieces of the scientific jigsaw fitted together resulting in the provision of chemotherapy to patients.

After nine years as a pharmacist, I started to feel competent in my role. Epstein and Hundert (2002) defined competence as “the habitual and judicious use of communication, knowledge, technical skills, clinical reasoning, emotions, the values and reflection in daily practice for the benefit of the individual and community being served”. I could understand how basic science knowledge had helped me grasp basic concepts. However, my undergraduate education had not provided the link with real life nor how the science could be appropriately applied to patient care. It took nine years of working in the health service to learn this. As a result of this experience, I felt I had found my niche as a researcher in medicine. A colleague with whom I was conducting the laboratory research advised that I study medicine and become a haematologist. The plan was to use my medical degree to forge a career in research.

In 2003, I went to Medical School in Norwich as a mature student. I was in the second cohort of a newly established medical school. My experience in Norwich was very different to that as a pharmacy undergraduate. I was introduced to a new method of learning. This being problem-based learning (PBL). Unlike the traditional method employed by medical schools in which students would undergo three years of didactic pre-clinical learning followed by two years of clinical placements, the PBL formula appeared to amalgamate the pre-clinical and clinical years together whilst providing focus towards a holistic approach to patient care. Unlike my experience as a pharmacy undergraduate, I felt I was learning through a combination of self-study, exploration, supportive lectures, direct patient contact at a general practice and hospital every week, and my PBL tutor. The course did not entirely focus on pure and applied science. There was also a focus on developing my learning skills, communication skills and essential practical skills that would prepare me for working as a doctor. The aim was that I would possess the skills to be committed to life-long learning in an ever-expanding world of science. This was in complete contrast to my experience as a pharmacy undergraduate. The beauty of the system at Norwich was that themes studied in one module were revisited in other modules within the course, thereby promoting spiral learning. This allowed me to remember and retain key concepts. I felt that my learning experience was not purely geared to passing exams.

However, I did feel that the depth of basic sciences taught on this course was not to the same level as that taught on traditional medicine courses. At the time I felt unsure as to why we were not being taught the intricacies of the Krebs cycle or why did I have to learn anatomy in a prosection classes. I wanted to learn anatomy in a traditional way, by which I would be assigned to a cadaver so I could spend hours dissecting and learning the structures of the human body. What I didn’t appreciate was that the course was devised such that we would study the anatomy and physiology of a specific region during the same module. For example, during the respiratory module, we would learn about the anatomy, physiology, biochemistry and pathology of the thorax. This would make it easy to relate to other modules such as cardiology and vascular medicine. I am not sure if the relationship between physiology, biochemistry and anatomy of an organ system was appreciated on a traditional course. The anatomy prosection classes to my surprise were excellent. In addition to learning the anatomy of a part of the body, the tutor related the anatomy to real-life application of that anatomy. For example, when interpreting chest x-rays, CT scans and MRI scans. Additionally meeting patients with respiratory pathology during the module allowed me to understand what was happening to them physiologically and how the multidisciplinary team was working together with the patient and their relatives in order to make accurate diagnoses and therapeutic plans. I quickly learnt that basic science formed a part of the global process in the management of the patient.

Whilst linking the basic sciences to body systems was useful for me, I was still not sure as to what depth of knowledge should be appropriate. Should I be learning Kreb’s cycle or not? The Kreb’s cycle would be useful for an endocrinologist or a specialist in metabolic medicine. Consider the following example, how the body forms haemoglobin should be linked to the haematology module with anaemia and other pathologies like porphyria. However, the depth of knowledge with regards to the production of haemoglobin should not be the same as that expected of haematology specialists. There is no point in a student understanding the intracellular signalling involved with the use of proteasome inhibitors in multiple myeloma at undergraduate level when they might have aspirations of being surgeons. I have also realised that this observation is also pertinent to anatomy. As an anaesthetist, I regularly perform regional anaesthesia for upper and lower limb surgery. I use ultrasound in order to perform these blocks. I would, therefore, require a better knowledge of anatomy than a student who is interested in public health. However, this understanding of applied anatomy can be acquired whilst undertaking my specialist training in anaesthesia. I felt confident that the basics I learnt during my prosection anatomy classes at medical school assisted with my learning of applied anatomy related to my clinical practice.

In an ever expanding field of science and expectations of doctors to be holistic practitioners, there is only so much content that can be assigned to a medical course. The University of East Anglia managed this by implementing a “student selected study project”. The purpose of this was to allow the student to select a subject from the syllabus from within prescribed domains, e.g. sociology, physiology, etc. The student could research a question of their own choice and present this to their peers at the end of a semester. This was done three times a year. The benefit was that the student could explore a range of areas of medicine that particularly interested them. The hope would be that the student would develop into a rounded individual, equipped with the tools for life-long learning.

For example, in one project, I chose to investigate the relationship between advertising and childhood obesity. I researched areas of psychology and sociology applied to medicine that I knew very little about. I looked at the reasons why obesity was an increasing problem and whether advertising bans would work. I applied my new found knowledge in sociological and psychological analysis to this area and discovered this to be an extremely complex subject, which involved social demographics and risk-taking behaviour. I found that advertising bans, *as a sole intervention,* did not work. From my research, I concluded that a bespoke tailored approach that identified the ideas, concerns, and expectations of the individual child is required. I found self-esteem to be the biggest indicator on how likely that individual was to engage in risk-taking behaviour, e.g. smoking or drinking alcohol ( Wild et al 2004). Additionally, behavioural and social factors are closely linked with childhood obesity ( Dehghan and Akhtar-Danesh 2005). I am very grateful to have had a learning experience in subjects that I do not think I would have even thought of as pertinent to medicine prior to starting medical school. Additionally, presenting three topics a year for five years to my peers developed self-confidence in me that I carry into my professional life. Unfortunately I did not experience this process of self-enlightenment whilst undertaking my Pharmacy degree. However, from talking to pharmacy undergraduates, I believe the way pharmacy is taught has also moved on from the traditional didactic methods I experienced a decade earlier. I visited my old School of Pharmacy and was impressed to see how much the syllabus has changed and how technology is used to enhance the student’s learning experience.

As I progressed through medical school and started work as a doctor, I realized that decisions my consultants were making and the decisions I am making now as an anaesthetic registrar are based on pattern recognition and evidence based practice as opposed to purely the use of basic science. This is especially noted when managing simple cases. However, when it came to managing complex cases, things are not as simple as “pattern recognition”

For example, I was involved in a case where a 72-year-old male experienced delayed emergence after anaesthesia following elective resection of an incidental asymptomatic meningioma ( Wilson-Baig et al 2016). When managing this patient, we had to rapidly consider possible causes that may have resulted in this situation whilst maintaining haemodynamic stability of the patient. This included anatomical, physiological, biochemical and pharmacological causes. We performed various investigations like a head CT scan to exclude an intracerebral bleed, blood glucose test to exclude hypoglycaemia, arterial blood gasses to exclude hypercarbia, hypoxia and acidosis and other related investigations. The whole assessment and initial investigations took around 45 minutes. No obvious cause for this was found, as all investigations were unremarkable. We had to use our knowledge of pure and applied sciences in a timely fashion to understand what was going on, instigate investigations and implement appropriate therapy whilst ensuring the safety of the patient. This case proved challenging as delayed emergence after anaesthesia is rare and little is known about it. There have not been enough cases to develop “pattern-recognition skills” to detect it as easily as a simple chest infection.

In order to make sense of what may have gone on, I reflected on the case and conducted my own literature review. This involved using my experience as a medical doctor and pharmacist. This experience was built upon on basic skills acquired at medical school, e.g. knowledge of the pure sciences, applied sciences, effective analyzing skills, the ability to interpret scientific data and partake in research. In the case described, frailty and genetic polymorphisms may have had an impact on the metabolism of anaesthetic agents ( McLachlan and Pont 2012, Watson et al 2012). This case demonstrated my transition from purely “understanding” the basic sciences at undergraduate level to independently combining this understanding with “action”, “analytical thinking”, “multidisciplinary team working”, and “continual learning”.

When it came to choosing a career, I found having an educational supervisor really helpful in my choice of a career in medicine. When I joined medical school, I wanted to become a haematologist. However, during my time as an undergraduate, I discovered anaesthesia. My elective with the Norfolk Air Ambulance and East Anglian Ambulance Services whetted an appetite in hospital and pre-hospital medicine. However, part of me still had a yearning to be involved in academia. Whilst at medical school and as an anaesthetist, I noted the difficulty of weaning ventilated patients who have Chronic Obstructive Pulmonary Disease (COPD) from a ventilator. I also noted a similar phenomenon in older “healthy” patients who had undergone prolonged ventilation. As a result, I developed an interest in the pathophysiological mechanisms involved in the ageing muscle, especially the diaphragm of patients undergoing continuous ventilation and in patients with COPD. Additionally, I also developed an interest in the stress response experienced by the critically ill patient. My long term plan is to investigate the physiological, psychological and psychosocial changes caused during the peri-operative and post-operative period. This, I appreciate is a challenge and I will come across obstacles. However, I have to be positive and find my own path in order to realise my ambitions. Successfully gaining a place as an NIHR Academic Clinical Fellow in Anaesthesia, has allowed me to develop research skills, whilst studying for an MSc in Clinical Research.

In summary, I believe that basic sciences at undergraduate level should be taught as an essential component in solving and managing complex cases. It should be integrated with other disciplines to encourage the student in becoming a holistic practitioner who has a thirst for life-long learning. Basic sciences at medical schools should prepare the student to further explore their chosen specialty at postgraduate level. My training at Norwich was first class and provided me with the skills to pursue my interests in research. My experience of studying two undergraduate degrees in two separate decades has shown me how much medical education has moved forward towards integrated learning with an aim to develop independent practitioners with an enquiring mind.

## Notes On Contributors

Dr Noamaan Wilson-Baig is an Anaesthetic Registrar (ST4) with Pennine Acute Hospitals NHS Trust. He is also an NIHR Academic Clinical Fellow. Noamaan has a background of fifteen years in Pharmacy with a specialist interest in Haematology, Oncology and Paediatric Pharmacy. Noamaan’s current interests are in Critical Care and Anaesthesia. His long-term plan is to pursue a career in academia with clinical practice. Twitter account @easinorm
