# Supplementary material for: The value of Life Sciences in an Integrated Curriculum: A Reflective Perspective of studying two Life Sciences Degrees a decade apart and the challenges faced in professional life
Source: MedEdPublish (2016). 2017 Jul 3;6:117. [Version 1] doi: 10.15694/mep.2017.000117 (PMC10885272; doi:10.15694/mep.2017.000117)
Supplement: Peer review history

# Reviewer report: Reviewer response for version 1

**DOI:** 10.21956/mep.19308.r28161

**Published:** 2019-04-04

**Authors:** Ken Masters

**Affiliations:** 1 Sultan Qaboos University

This review has been migrated. The reviewer awarded 4 stars out of 5 An interesting read, in which the author compares his experiences of undergoing a more traditionally-structured undergraduate degree to an integrated degree course. The literature and personal experience certainly support the author’s experience of PBL. (It would be difficult to know how much the author’s prior knowledge from their first degree, experience and maturity impacted on his ability to learn and understand the information in the second.) Nevertheless, the author has provided rare and valuable insight from his dual experience, and this paper would be a useful read for those responsible for curriculum construction at medical schools that still adhere to the old-style approach.

Reviewer Expertise:

NA

No decision status is available

# Reviewer report: Reviewer response for version 1

**DOI:** 10.21956/mep.19308.r28160

**Published:** 2017-07-18

**Authors:** John Dent

**Affiliations:** 1 AMEE

This review has been migrated. The reviewer awarded 4 stars out of 5 It is always interesting to read a personal reflection on medical education - especially when it is making such an enthusiastic endorsement of the advantages of a reformed curriculum.In this paper the author compares his initial traditional training and subsequent employment in pharmacy to his recent experience in a new medical school with a reformed curriculum and his current clinical practice. He reports that competency-based education was not a component of his original degree course and that he felt unprepared for the realities of working-life as a pharmacist.In a later research degree he appreciated the contribution of activity and task-based learning and the importance of integration. Some years later at medical school he encountered a reformed curriculum with a problem-based approach to learning and the longitudinal and horizontal integration of basic sciences. He found that these disciplines contributed an essential component in solving and managing complex cases and that the integration of these disciplines within the curriculum can encourage students in becoming holistic practitioners with a capacity for directed self-learning.Interestingly, although not quoted in the references, these personal reflections chime with an approach to teaching and learning described by Harden & Laidlaw (2013); four principles that lead to more effective learning, Feedback, Active learning, Individualisation and Relevance. His reflection also resonates with the SPICES approach to a reformed curriculum described by Harden, Sowden and Dunn (1984) as being Student-centred, Problem based, Integrated, Community –based, having Electives and being Systematic in approach. It is interesting how the author’s personal experiences have led to some of these same ideas.In this personal reflection the author’s endorsement of a reformed curriculum, encapsulated in his closing comment, could be a mantra for other medical schools to follow, “Medical education has moved forward towards integrated learning with an aim to develop independent practitioners with an enquiring mind”. References:Harden RM & Laidlaw JM. 2013. Be FAIR to students: Four principles that lead to more effective learning, Med Teach 35:27-31.Harden RM, Sowden S, and Dunn WR. 1984. Educational strategies in curriculum development: the SPICES model. Med Educ 18:284-297.

Reviewer Expertise:

NA

No decision status is available
